# Silencing of *GLS* and overexpression of *GLS2* genes cooperate in decreasing the proliferation and viability of glioblastoma cells

**DOI:** 10.1007/s13277-013-1247-4

**Published:** 2013-10-06

**Authors:** Monika Szeliga, Małgorzata Bogacińska-Karaś, Aleksandra Różycka, Wojciech Hilgier, Javier Marquez, Jan Albrecht

**Affiliations:** 1 Department of Neurotoxicology, Mossakowski Medical Research Centre Polish Academy of Sciences, 5 Pawińskiego Str., 02-106 Warsaw, Poland; 2 Faculty of Horticulture, Biotechnology and Landscape Architecture, Warsaw University of Life Sciences, 166 Nowoursynowska Str., 02-787 Warsaw, Poland; 3 Department of Molecular Biology and Biochemistry, Faculty of Sciences, Campus de Teatinos, University of Málaga, 29071 Málaga, Spain

**Keywords:** *GLS*, *GLS2*, Proliferation, Viability, Glioblastoma

## Abstract

Glutamine (Gln) metabolism, initiated by its degradation by glutaminases (GA), is elevated in neoplastic cells and tissues. In malignant glia-derived tumors, GA isoforms, KGA and GAC, coded by the *GLS* gene, are overexpressed, whereas the *GLS2*-coded GAB and LGA isoforms, are hardly detectable in there. Our previous study revealed that transfection of T98G glioblastoma cells with GAB reduced cell proliferation and migration, by a yet unknown mechanism not related to Gln degradation. The question arose how simultaneous overexpression of GAB and inhibition of KGA would affect glioblastoma cell growth. Here, we used siRNA to silence the expression of Gls in T98G cells which were or were not stably transfected with GAB (TGAB cells). In both T98G and TGAB cell lines, silencing of Gls with siRNAs targeted at different sequences decreased cell viability and proliferation in a different, sequence-dependent degree, and the observed decreases were in either cell line highly correlated with increase of intracellular Gln (*r* > 0.9), a parameter manifesting decreased Gln degradation. The results show that combination of negative modulation of GA isoforms arising from *GLS* gene with the introduction of the *GLS2* gene product, GAB, may in the future provide a useful means to curb glioblastoma growth in situ. At the same time, the results underscore the critical role of Gln degradation mediated by KGA in the manifestations of aggressive glial tumor phenotype.

## Introduction

Malignant gliomas are the most common brain tumors in adults which despite diverse therapeutic efforts remain untreatable [1]. Hallmarks of glioma include high proliferation, diffuse invasion, and resistance to chemo- and radiotherapy.

Glutamine (Gln) is a central metabolite in many neoplastic cells and tissues, including gliomas, and an increased glutaminolysis is a characteristic feature of tumors, irrespective of their tissue origin [2]. Glutaminase (GA; EC 3.5.1.2) is the enzyme catabolizing most of the tissue Gln to glutamate (Glu) and ammonia. Two genes coding for GA have been identified: the *Gls* gene encodes the kidney-type isoforms (KGA and GAC) and the *Gls2* gene encodes the liver-type isoforms (LGA and GAB) [3]. KGA is expressed in all mammalian tissues except liver [4], while its alternatively spliced variant, GAC, was found in the heart, pancreas, kidneys, lungs, and breast cancer cells [5]. There are also two transcripts arising from the *Gls2* gene: LGA is expressed in liver [4] and GAB is expressed in the brain, pancreas, leukaemic cells [6], breast [7], and colorectal carcinoma cells [8]. Recently, it has been reported that GAB and LGA are coexpressed in mammalian brain and liver by using an alternative transcription initiation mechanism and alternate promoters [9]. Nuclear localization of GAB protein in the central nervous system and its interactions with other proteins suggest that the physiological role of this isoform may go beyond GA activity [10, 11].

Elevated glutaminolysis in cancer cells involves altered expression and/or activity of GA isoforms [12]. The expression pattern of distinct GA isoforms in several human-derived neoplastic cell lines and tissues allows hypothesizing that isoforms encoded by *GLS* are upregulated in parallel with the proliferation rate, whereas isoforms encoded by *GLS2* are related to a quiescent, non-proliferating, cell state [6]. Noteworthy, *GLS* overexpression induced by the oncogene *c*-Myc via miR-23 suppression is related to enhanced cell proliferation [13]. Silencing of *GLS* significantly decreased proliferation of prostate cancer cells in vitro [13], Ehrlich ascites tumor cells in vitro and in vivo [14], and T98G glioblastoma cells [15]. By contrast, *GLS2* is a target gene of tumor suppressor p53 and plays a pivotal role in mediating the functions of p53 in energy metabolism and antioxidant defense [16]. Overexpression of *GLS2* in hepatocellular carcinoma cells reduced cell growth and colony formation [16, 17].

In glioblastomas (WHO grade IV), the most malignant brain tumors, high levels of *GLS* and only traces or lack of *GLS2* transcripts were found [18]. Likewise, human glioblastoma T98G cell line expresses high amounts of *GLS* transcripts, while *GLS2* transcripts are hardly detectable in these cells. Transfection of T98G cells with a GAB cDNA sequence diminished cell proliferation and survival [19].

An intriguing question arose whether or not combination of *GLS* silencing and *GLS2* overexpression would increase the inhibition of cell proliferation and survival of glioblastoma cells elicited by individual manipulations. To answer this question, the expression of KGA and GAC isoforms was knocked down in a human glioblastoma cell line that was (TGAB cells) or was not (T98G cells) previously transfected with GAB cDNA, respectively [19], and the two parameters describing the development of glioma were investigated in so treated cells. We employed graded inhibition of KGA and GAC in both T98G and TGAB cells to analyze the correlation between the phenotypic changes and the Gln content of the cells as a marker of the intensity of its consumption.

## Materials and methods

### Cell lines and culture conditions

T98G human glioblastoma cell line purchased from American Type Culture Collection and their derivative TGAB were maintained in minimum essential medium (Sigma-Aldrich) supplemented with 10 % FBS, 1 % nonessential amino acids, and 1 % antibiotics (penicillin and streptomycin). Cultures were maintained at 37 °C in a humidified atmosphere with 95 % air and 5 % CO_2_. The culture medium for TGAB cell lines containing the neomycin-resistance gene was supplemented with 0.5 mg/ml G418 (Sigma-Aldrich). The expression of the *Gls2* gene in both cell lines was monitored by RT-PCR as described previously [19].

### Construction of siRNAs

Silencer siRNA Construction Kit (Ambion) was used to design and construct siRNAs. Briefly, five target sequences (Table 1) within the human *Gls* mRNA sequences (GenBank accession no.: NM_014905.4 and NM_001256310.1 for KGA and GAC transcript, respectively) were chosen according to the manufacturer’s protocol. All the chosen sequences contain less than 17 contiguous base pairs of homology to other coding sequences within the human genome. The sense and antisense template DNA oligonucleotides for each of five siRNAs (termed siGls3–7) were synthesized (IBB, PAS) and used for in vitro transcription. Obtained siRNAs were purified and quantified with NanoDrop 2000 UV/Vis Spectrophotometer. To control for nonspecific events, scrambled sequence oligonucleotides (scr) with the same base composition as the antisense oligonucleotide, but in arbitrary order, were employed.

**Table 1:** Sequences targeted by anti-Gls siRNA

siRNA	Target sequence	Position in NM_014905.4 and NM_001256310.1
siGls3	AAGAGTGTATGGATATGTTA	791–811
siGls4	AAGTGCTAAAAAGCAGTCTGG	972–992
siGls5	AAGTTCCCTTCTGTCTTCAGT	1,100–1,120
siGls6	AATGGTGGTTTCTGCCCAATT	1,570–1,590
siGls7	AACTATGATAATTTGAGACAC	1,849–1,869

GenBank accession numbers NM_014905.4 and NM_001256310.1 refer to KGA and GAC transcripts, respectively

### Transient transfection

Transient transfection of T98G cells and TGAB cells was performed with Lipofectamine 2000 (Invitrogen), according to the manufacturer’s protocol. Briefly, the day before transfection, cells were seeded in antibiotic-free growth medium for 24 h. Next, complexes containing Lipofectamine and siGls3–7 or scr were directly added into the media. The knockdown of *GLS* was measured by quantitative real-time PCR and Western blot 48 h after transfection.

### RNA isolation and RT-PCR

Total RNA from the transfected cells was extracted using a guanidinium-thiocyanate-based commercial kit (TRI-Reagent, Sigma). Two micrograms of RNA was digested with DNaseI (Invitrogen) and reverse-transcribed using a High-Capacity cDNA Reverse Transcription Kit (Applied Biosystems) according to the manufacturer’s protocol.

### Real-time PCR

Real-time PCR analyses were conducted using TaqMan Gene Expression Assays and TaqMan Universal PCR MasterMix (Applied Biosystems) according to the manufacturer’s protocol. The assays IDs are listed in Table 2. The reactions were incubated in a 96-well optical plates at 95 °C for 10 min, followed by 45 cycles of 95 °C for 15 s and 60 °C for 1 min using an Applied Biosystems 7500 Sequence Detection System. Relative expression was calculated using the ΔΔC_T_ method [20] and normalized to the expression β-actin.

**Table 2:** TaqMan gene expression assays used for real-time PCR

Transcript	Assay ID	GeneBank sequence	Exon boundary
KGA	Hs01014019_m1	NM_014905.4	17–18
GAC	Hs01022166_m1	NM_001256310.1	14–15

### Western blot analysis

The cells were harvested and sonicated in radioimmunoprecipitation assay lysis buffer supplemented with cocktails of protease and phosphatase inhibitors (Sigma-Aldrich). Lysates were centrifuged at 12,000×*g* for 10 min at 4 °C. Protein concentration was determined with BCA Protein Assay Kit (Thermo Scientific Pierce). Samples of 30 μg total protein were subjected to sodium dodecyl sulfate (SDS)-PAGE. Resolved proteins were transferred to nitrocellulose membrane and probed with specific primary antibodies: anti-Gls or anti-GAC (Proteintech) and secondary antibody conjugated to horseradish peroxidase (Sigma-Aldrich). Blots were developed with SuperSignal West Pico chemiluminescence substrate (Thermo Scientific Pierce). For loading control, membranes were stripped and reprobed with a human-specific antibody against GAPDH (Sigma-Aldrich). Densitometric analysis was done using G:Box system and GeneTools software (Syngene).

### Intracellular Gln and Glu assays

Gln and Glu in cell supernatants were determined using HPLC as described previously [19]. Briefly, 48 h post-transfection, T98G and TGAB cells were washed with PBS, incubated in 5-sulfosalicylic acid for 5 min and centrifuged at 12,000×*g* for 5 min. The level of amino acids in supernatants was analyzed using HPLC with fluorescence detection. Protein content was measured in the supernatants using the method described by Bradford [21].

### Cell viability

Cell viability was determined by means of a 3-(4,5-dimethylthiazol-2-yl)-2,5-diphenyl tetrazolium bromide (MTT) conversion as described previously [19]. Briefly, equal numbers of T98G and TGAB cells (4 × 10^4^/well) were seeded in 24-well plates and incubated for 24 h. After this time, the cells were transfected with siGls3–7 or scr siRNA and cultured for further 48 h. Next, the medium was removed and the cells were washed with PBS and incubated in the culture medium with MTT solution at the final concentration of 0.5 mg/ml for 15 min to allow the conversion of MTT into formazan. Then, the medium was replaced with 5 % SDS and absorbance was read at 570 nm using Elisa Bio-Rad Microplate Reader. Cell viability was expressed as a mean of four replicate wells from four independent experiments.

### Cell proliferation assay

DNA synthesis as a marker for cellular proliferation was measured by bromodeoxyuridine (BrdU) incorporation using the Cell Proliferation Elisa BrdU (colorimetric) assay (Roche) according to the manufacturer’s instruction. Briefly, equal numbers of T98G and TGAB cells (5 10^3^/well) were seeded in 96-well plates and incubated for 24 h. After this time, the cells were transfected with siGls3–7 or scramble siRNA and cultured for further 48 hours. Next, BrdU was added for 2 h, and then cells were fixed for 30 min and incubated with BrdU antibody for 90 min. The absorbance values were measured at 415 nm using Elisa Bio-Rad Microplate Reader. Cell proliferation was expressed as a mean of four replicate wells from six independent experiments.

### Statistical analysis

All experiments described above were repeated at least four times. Results are reported as means and standard deviations. Statistical significance of comparisons was based on one-way ANOVA followed by Tukey’s test.

## Results

**Fig. 1:**
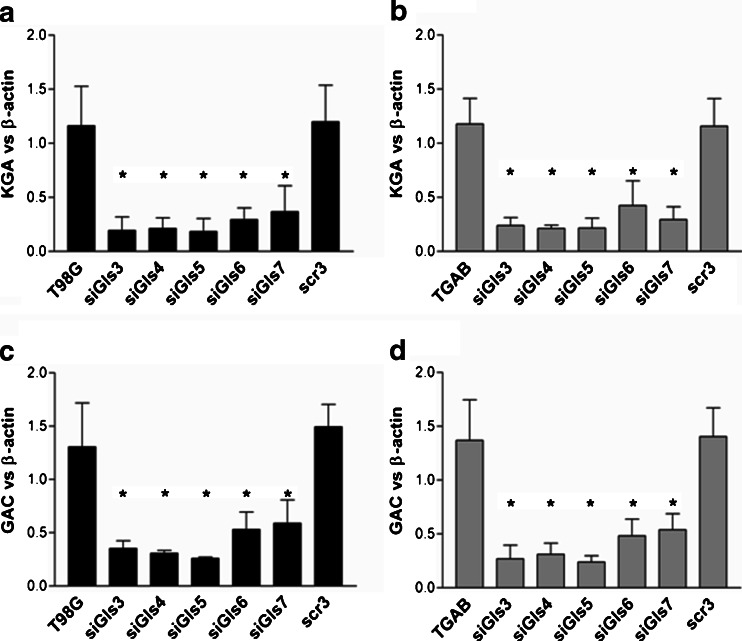
Silencing of *GLS* decreases the level of KGA and GAC transcripts. Relative expression of KGA (**a**, **b**) and GAC (**c**, **d**) transcripts measured vs beta-actin expression. The results are mean ± SD for five mRNA isolations from each cell line. *p < 0.01 vs untreated or treated with scrambled siRNA cells as tested with one-way ANOVA followed by Tukey’s test

To explore the role of GLS GA isoforms in T98G and TGAB cells, we transiently knocked down both transcripts arising from the *GLS* gene using five siRNAs targeting KGA and GAC sequences in different sites. The primary assumption was that all five siRNAs will silence both transcripts arising from the *GLS* gene with equal potency. The level of KGA transcript was decreased by more than 80 % in case of treatment with siGls3–5 and more than 70 % in case of treatment with siGls6 and siGls7 (Fig. 1a, b). The reduction of GAC transcript level was less pronounced and ranged from approx. 80 % in case of treatment with siGls3–5 and approximately 60 % in case of treatment with siGls6 and siGls7 (Fig. 1c, d). There was no significant difference between the nontransfected cells and the cells transfected with scr siRNA indicating that the transfection process itself had no influence on the *GLS* expression. As no differences were observed between the effectiveness of distinct scrambled siRNAs, only the results obtained for scrambled oligonucleotide 3 (scr3) are illustrated.

**Fig. 2:**
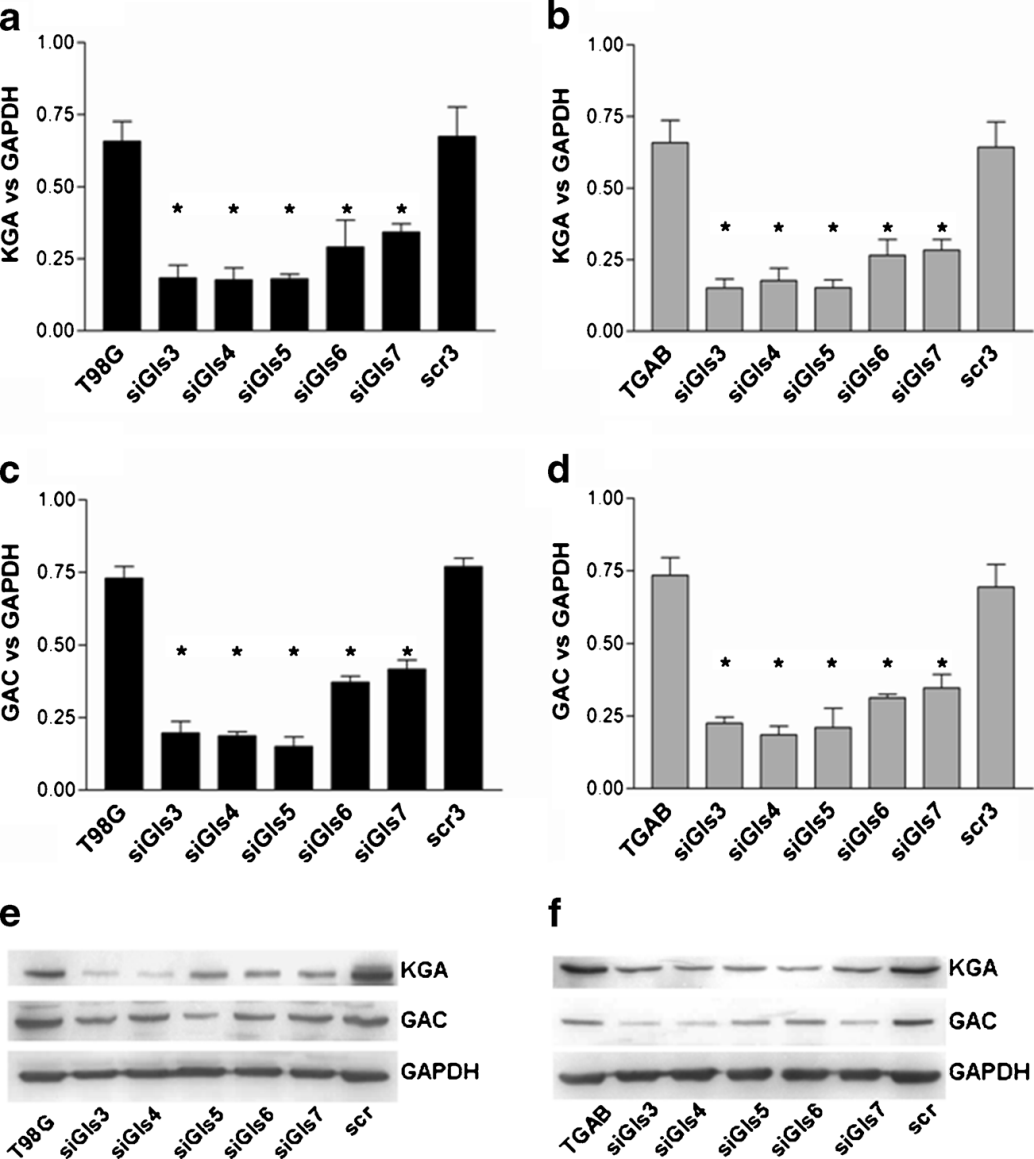
Silencing of *GLS* decreases the level of KGA and GAC proteins. The graphs show quantification of KGA (**a**, **b**) or GAC (**c**, **d**) band intensity normalized to GAPDH. The results are mean ± SD for four protein isolations from each cell line. *p < 0.01 vs untreated or treated with scrambled siRNA cells as tested with one-way ANOVA followed by Tukey’s test. In the lowest part, representative Western blots show amounts of KGA, GAC, and GAPDH in protein extracts from T98G (**e**) or TGAB (**f**) cells treated with siGls3–7

Western blot analyses, using antibodies specific to the KGA or GAC isoform, showed a decreased levels of either protein in T98G (Fig. 2a, c, e) and TGAB (Fig. 2b, d, f) cell lines. Again, siGls3-5 presented higher effectiveness than siGls6 and siGls7.

**Fig. 3:**
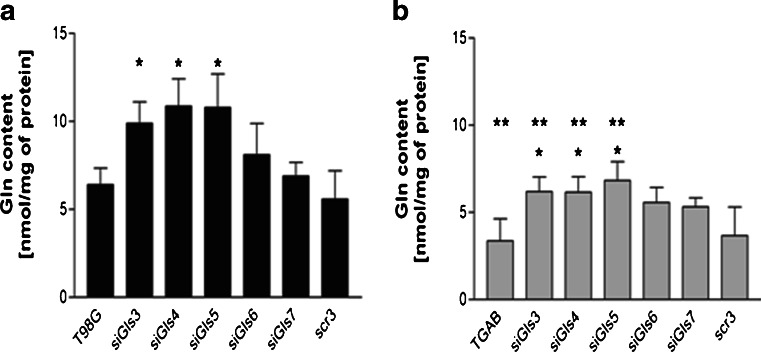
Silencing of *GLS* increases intracellular level of Gln in T98G (**a**) and TGAB (**b**) cells. T98G and TGAB cells were transfected with siGls3–7. The level of intracellular Gln was measured at 48 h post-transfection. The results are mean ± SD for four independent determinations for each cell line. *p < 0.05 vs untreated cells and treated with scrambled siRNA (*scr3*); **p < 0.05 vs T98G counterparts as tested with one-way ANOVA followed by Tukey’s test

Next, we evaluated the effects of the presence or absence of each GA on the intracellular levels of Gln content in the differently transfected cells. Consistent with our previous study [19], cells enriched in GLS2 (TGAB) contained ∼2 times less Gln and than T98G cells (Fig. 3). In both cell lines, reduction of GLS content by treatment with siGls3–5 increased Gln level, while the effects of siGls6 and siGls7 treatments on Gln level were insignificant (Fig. 3). Of note, in TGAB cells treated with siGls3–5 the intracellular Gln amount was significantly lower than in T98G counterparts. No differences in Gln levels were observed between untreated cells and cells treated with scr-RNA. Glu levels decreased insignificantly after treatment with each of the five siRNAs (data not shown; Glu level was in all cases present in a manifold excess of Gln).

**Fig. 4:**
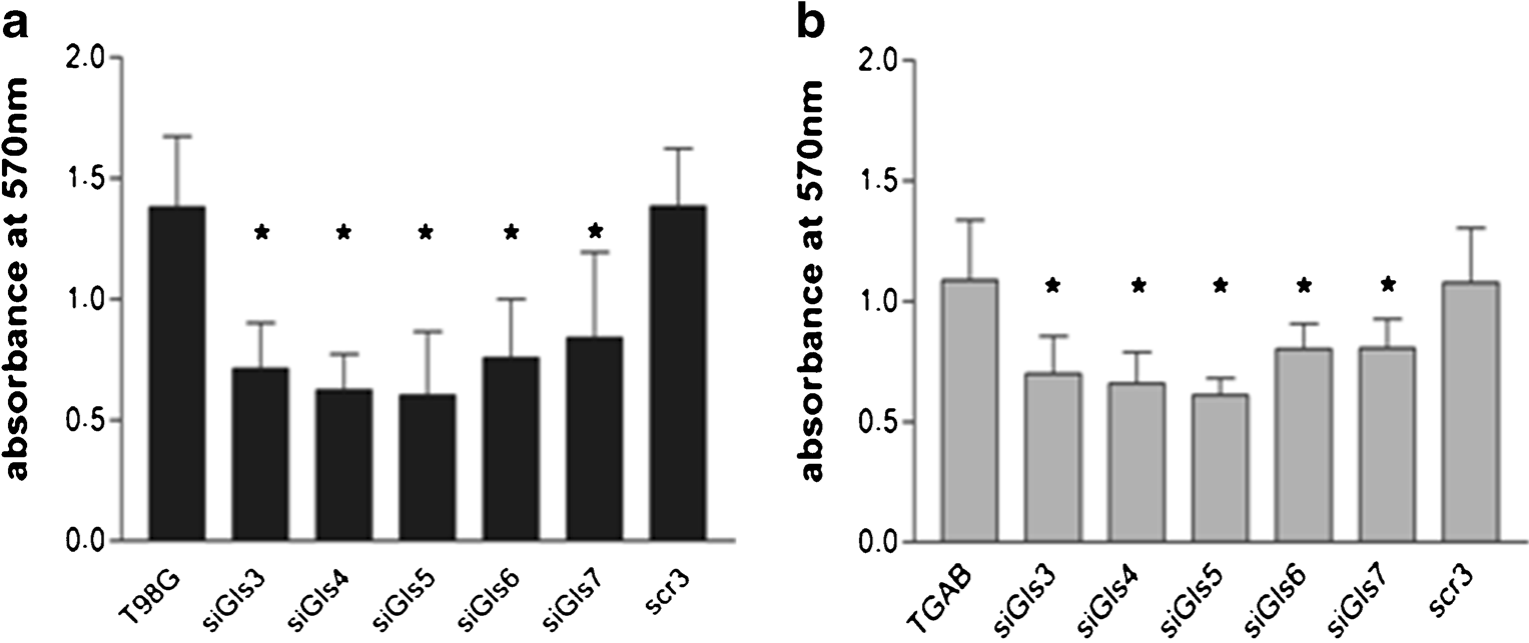
Silencing of *GLS* decreases survival of T98G (**a**) and TGAB (**b**) cells. T98G and TGAB cells were transfected with siGls3–7. Mitochondrial activity was assessed by the MTT test at 48 h post-transfection. The results are mean ± SD for four independent determinations for each cell line. *p < 0.05 vs untreated cells and treated with scrambled siRNA (*scr3*) as tested with one-way ANOVA followed by Tukey’s test

MTT test showed a significant reduction of viable cell mass following *GLS* knockdown by siRNA treatment in both cell lines (Fig. 4). Treatment of T98G and TGAB cells with siGls3–5 decreased cell viability by ∼50 and ∼65 %, respectively, while in the case of siGls6 and siGls7 the reduction amounted to ∼40 and ∼20 % in T98G and TGAB cells, respectively. No differences in cell viability were observed between cells treated with scr-RNA and untreated cells.

**Fig. 5:**
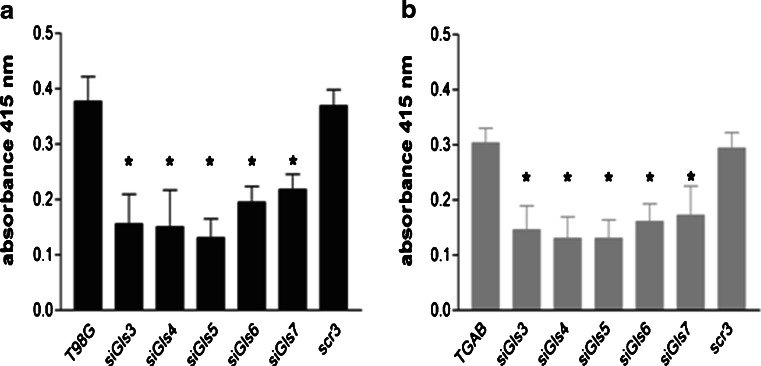
Silencing of *GLS* decreases proliferation of T98G (**a**) and TGAB (**b**) cells. T98G and TGAB cells were transfected with siGls3–7. Proliferation was assessed by the BrdU test at 48 h post-transfection. The results are mean ± SD for six independent determinations for each cell line. *p < 0.05 vs untreated cells and treated with scrambled siRNA (*scr3*) as tested with one-way ANOVA followed by Tukey’s test

To examine the influence of the GLS knockdown on cell proliferation, BrdU incorporation was monitored in T98G and TGAB cells after treatment with siGls3–7. In contrast to untreated counterparts and cells treated with scr siRNA, the percentage of BrdU-incorporated cells was reduced by nearly 65 % in T98G and 60 % in TGAB cells after transfection with siGls3–5, respectively, while treatment with Gls6 and Gls7 decreased proliferation of both cell lines by ∼40 % (Fig. 5a, b). Again, the values of absorbance were lower in case of TGAB cells than in case of T98G cells.

**Fig. 6:**
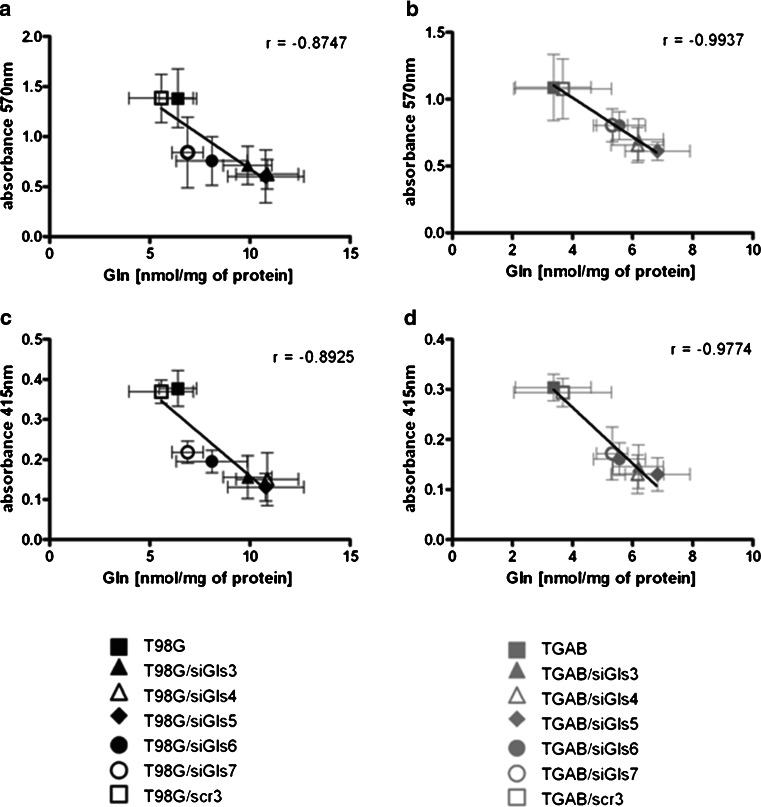
Correlations between Gln level and cell viability or proliferation. **a** Correlation between Gln level and results of MTT assay in T98G cells (Pearson’s correlation coefficient: *r* = −0.8747, *p* = 0.01). **b** Correlation between Gln level and results of MTT assay in TGAB cells (*r* = −0.9937, *p* < 0.0001). **c** Correlation between Gln level and results of BrdU assay in T98G cells (*r* = −0.8925, *p* < 0.01). **d** Correlation between Gln level and results of BrdU assay in TGAB cells (*r* = −0.9774, *p* = 0.0001)

Next, we studied how the phenotypic characteristics of cells treated in different GA-modifying paradigms correlated with the intracellular Gln content evoked by the treatments. In both cell lines, viable cell mass negatively correlated with the level of intracellular Gln (*r* = −0.8747, *p* < 0.05; *r* = −0.9937, *p* < 0.0001 for T98G and TGAB, respectively) (Fig. 6a, b). Similarly, proliferation negatively correlated with the level of intracellular Gln (*r* = −0.8925, *p* < 0.01; *r* = −0.9774, *p* = 0.0001 for T98G and TGAB, respectively) (Fig. 6c, d).

## Discussion

Elevated Gln metabolism fulfills the demands of increased proliferation and growth displayed by neoplastic cells of different origin [2]. Consistently, upregulated expression and activity of GA catabolizing Gln is a hallmark of these cells [12]. Studies conducted during the past decade have demonstrated that GA isoforms play opposite roles in tumorigenesis. In the different native neoplastic cells or cultured cell lines examined so far, the GLS isoforms are associated with cell proliferation, whereas the GLS2 isoforms are attributed to resting or quiescent cell states [6, 8]. Knocking down of *GLS* gene in the mouse mammary tumor cells [14], the MCF-7 breast cancer cells [22], and glioblastoma cells [15] led to a reversion of the transformed phenotype. Similar phenotype reversion was attained by overexpression of the *GLS2* gene in nonsmall-cell lung carcinoma cells [17], while exogenous GLS2 expression reduces cell colony formation in human hepatocellular carcinoma and lung cancer cell lines [16]. Likewise, our previous studies revealed that stable transfection of a full cDNA sequence encoding GAB protein (a GLS2 isoform) reduces proliferation and migration of glioblastoma T98G cell line [19] and sensitizes transfected cells (TGAB cells) to the alkylating chemotherapeutics [23].

Here, we used five siRNAs complementary to different sites within mRNA for KGA and GAC transcripts arising from *GLS* gene. Three of these siRNAs inhibited the expression of both transcripts with ∼80 % efficiency, while two of them reduced the expression by ∼60 %. The reasons for discrepancy between the potency of distinct siRNAs are unknown. Most likely, it results from differences in target sequence accessibility, as some regions of mRNA may be highly structured or bound by some proteins [24].

In this study, *GLS* silencing significantly decreased viability and proliferation of glioblastoma T98G cells which do not express appreciable amounts of GA isoforms arising from *GLS2* gene. This effect is likely to be representative for other glioblastoma-derived cell lines or glioblastomas that possess relatively low levels of the *Gls2* gene product. The level of viability and proliferation highly negatively correlated with the level of intracellular Gln, reinforcing the importance of this amino acid for growth of glioblastoma cells. These results are consistent with data obtained by other groups [15, 25].

To our knowledge, this is the first study documenting the effect of *GLS* silencing in glioblastoma cells expressing substantial amount of GAB (TGAB cells). Similarly to T98G cells, TGAB cells treated with anti-GLS siRNA displayed significant decrease in proliferation and viability and both parameters were negatively correlated with the level of intracellular Gln. The basic function of proteins encoded by both *GLS* and *GLS2* genes is to metabolize Gln. Therefore, lack of any GA isoform resulting in uppermost intracellular Gln level should diminish cell growth most effectively, while graded decrease in Gln level should entail increase of cell growth. However, it was not exactly the case in this study. In spite of significantly lower level of intracellular Gln, TGAB cells displayed reduced growth as compared to T98G cells. Moreover, TGAB cells treated with anti-GLS siRNA presented lower proliferation and viability, although the level of intracellular Gln in these cells was significantly diminished as compared to T98G so treated. This results support the earlier observations that GLS2 isoforms play opposite role to GLS isoforms with regard to promotion of cell proliferation [6, 8]. Of note, the proportional decrease of viability and proliferation evoked by *GLS* silencing was higher in T98G than in TGAB cells. This last result is consistent with the observation TGAB cells overexpressing GLS2 already acquired a more differentiated and less malignant phenotype [19].

The physiological meaning of simultaneous existence of GA isoforms in one cell type remains unknown. However, there is a growing body of evidence that proteins arising from these two genes are involved in different signaling pathways. The hypothesis that GAB is a multifunctional protein fulfilling roles other than enzymatic is originally based on finding that this isoform can interact with PDZ-containing proteins [26] and may be localized in the neuronal nuclei [10]. Later studies showed that *GLS2* is a target gene of p53 tumor suppressor to mediate the role of p53 in cellular energy metabolism and antioxidant defense mechanisms [16, 17]. Very recent findings confirmed more significant role of GAB than KGA in controlling redox status in neoplastic cells [25, 27]. On the contrary, *GLS* gene has been demonstrated to be regulated by *c*-Myc oncogene and upregulated in parallel with cancer cell proliferation [13].

In conclusion, the key finding of this study is that *GLS* silencing reduces proliferation and viability of glioblastoma cells and strengthen the antiproliferative effect evoked by GLS2 overexpression. As such, the finding underscores the critical role of Gln degradation in the manifestation of aggressive glial tumor phenotype. From the clinical perspective, it is to be hoped that that combination of modulation of expression and/or activity of GA isoforms arising from *GLS* and *GLS2* gene products may in the future provide a useful treatment modality to prevent glioblastoma growth. We want also to stress the fact that glioblastoma TGAB cells overexpressing GLS2 are more susceptible to chemotherapy with alkylating agents [23].

A more detailed analysis of this phenomenon is currently conducted in our laboratory with derivatives of T98G and TGAB cells stably transfected with a vector carrying anti-GLS sequence. Preliminary experiments indicate that until the first passage T98G cells transfected with such a vector proliferate very slowly, while after the first passage their proliferation increases, although it is still significantly reduced as compared to the non-transfected counterparts. This observation could be a consequence of a compensatory anaplerotic mechanism described by Cheng et al. [15], allowing the cells to use glucose instead of Gln. Interestingly, a study of tumor metabolism in vivo using an orthotopic mouse model of primary human glioma revealed that the tumors and surrounding brain showed metabolic differences, notably the accumulation of a large Gln pool within the tumors [28]. On the contrary, TGAB cells transfected with a vector carrying anti-GLS sequence present strongly diminished, but more stable level of proliferation suggesting that they do not have to adapt to Gln-free conditions.
